# Study of electrocardiographic peculiarities associated with elite football refereeing in Burkina Faso

**DOI:** 10.17159/2078-516X/2025/v37i1a23667

**Published:** 2025-12-15

**Authors:** A Tiama, A Sawadogo, AJF Tiendrébeogo, A Kaboré, AR Cissé, B Nana, NV Yaméogo

**Affiliations:** 1 Institute of Sports Sciences and Human Development, Joseph Ki-Zerbo University, Burkina Faso; 2 Department of Pulmonology, Yalgado Ouédraogo University Hospital, Joseph Ki-Zerbo University, Burkina Faso; 3 Department of Cardiology, Yalgado Ouédraogo University Hospital, Joseph Ki-Zerbo University, Burkina Faso

**Keywords:** central and assistant referees, electrocardiogram, athlete’s heart

## Abstract

**Background:**

Knowledge of the electrical characteristics of the athlete’s heart is essential for differential diagnosis with latent cardiac pathologies at risk.

**Objectives:**

This study aimed to determine the electrical characteristics induced by refereeing in football.

**Methods:**

This was a cross-sectional study conducted with 57 Burkinabe elite referees divided into two groups: the central referees’ group and the assistant referees’ group. The recorded ECGs were interpreted according to the 2017 expert consensus criteria.

**Results:**

PR and QT intervals, then the Sokolow Lyon index of the left ventricle, were increased more (p < 0.05) in the central referees’ group than in the assistant referees group. Resting heart rate and corrected QT interval were lower (p < 0.05) in central referees. The number of referees with sinus bradycardia (23 vs 18; p = 0.04), early repolarisation (16 vs 9; p = 0.03), incomplete right bundle branch block (14 vs 6; p = 0.02) and left ventricular electrical hypertrophy (17 vs 11; p = 0.03) were higher in the central referees group than in the assistant referees group.

**Conclusion:**

The results indicated that football refereeing was associated with myocardial electrical adaptations, but these adaptations are more frequent in central referees. An evaluation of Doppler echocardiography parameters is recommended to complement these findings.

In football, referees are essential players because they ensure the smooth progression of matches by applying the laws of the game and protecting players’ physical safety.^[1]^ Referees must keep pace with the game to be in the right position at the right time to make appropriate decisions.^[2]^ This task, assigned to referees, requires good endurance, which depends on the heart’s performance.^[1]^

The analysis of the cardiac muscle of football referees has attracted scientific interest in recent years.^[3–7]^ In Italy, a strong correlation is observed between maximal oxygen consumption (V̇O_2_max) and left ventricular internal diameters of elite referees.^[3]^ Another study found that the left ventricular mass index of these referees was higher than that of amateur football players but lower than that of professional football players.^[5]^ In Spain, the systolic and diastolic functions recorded in elite referees were normal, and the left ventricular mass was more developed than that of the general population. However, the left ventricular mass of these referees was less developed than that of professional footballers.^[5]^

A study conducted in Benin showed that elite and sub-elite football referees have normal cardiac morphology and function or those associated with the athlete’s heart.^[6]^ After 10 weeks of refereeing activities during competition, this study did not observe significant changes in cardiac morphology and function in the group of elite and sub-elite referees studied.^[6]^ Work carried out with 121 Burkinabe referees identified seven with an abnormal resting ECG profile, but cardiovascular parameters during the exercise test did not reveal any latent cardiac pathologies.^[7]^

Regular and long-term practice of intensive physical activity causes changes in the resting electrocardiogram (ECG) that reflect the physiological adaptation of the heart to training.^[8]^ These changes are the electrical characteristics of the athlete’s heart and vary according to sport, sex, and ethnic origin. In the absence of a positive family history for heart disease or sudden cardiac death, and a clinical examination, these do not require additional examinations in asymptomatic athletes.^[8]^ Therefore, their knowledge is essential for the differential diagnosis of latent cardiac pathologies at risk, including hypertrophic cardiomyopathy, the main cause of sudden death in athletes under 35 years of age.^[8]^

In sub-Saharan Africa, the electrical specificities of the athlete’s heart have been studied in footballers in Ghana, Mali and Burkina Faso.^[9–11]^ On the other hand, there are no related studies on football referees, even though, since 2010, resting ECG has been made mandatory for referees by the International Federation of Association Football to prevent sudden death.^[12]^ This is why we found it necessary to carry out this study, which aims to: 1) determine the electrical specificities of the athlete’s heart in Burkinabe central and assistant football referees, and 2) compare the electrical specificities of central referees with those of assistant referees.

## Methods

This was a descriptive cross-sectional study conducted with Burkinabe FIFA- or federal-level football referees recognised by the Central Referees Commission (CRC) of Burkina Faso Football Federation (FBF). Authorization from the Central Referees Commission (CRC)/Burkina Faso Football Federation (FBF) was obtained before the study was carried out.

### Participants

The study population consisted of elite Burkinabe male football referees selected from the official CCA/FBF list to officiate the Burkinabe first division championship for the 2024–2025 season. To participate in the study, participants had to be central or assistant referees with at least 12 years of refereeing experience, including 5 years in the first division. Thus, 57 referees, including 10 FIFA and 47 federal referees, volunteered to participate in the study. These referees were divided into two groups, including a Central Referees’ Group (CRG: n = 28) made up of 4 FIFA and 24 Federal referees and an Assistant Referees’ Group (ARG: n = 29) made up of 6 FIFA and 23 Federal referees.

### Ethical considerations

Before the study began, an interview was conducted with the referees regarding the study process. The referees also obtained prior assurance that the collected data would be used confidentially, anonymously, and exclusively for this study before each provided their free, informed, and written consent.

### Measurements

Referees’ height was measured using a wall-mounted body meter (Seca 206, France). A multifunction electronic bioimpedance meter (Terraillon, China) was used to measure body mass (BM), body fat percentage (%BC) and Body mass index (BMI). Blood pressure was measured manually using a Manopoire Spenler blood pressure monitor (Lian Sonic, France). V̇O_2_ max was indirectly estimated based on Yo-Yo IRT1 performance using the formula V̇O_2_ max (ml·min^−1^·kg^−1^) = Distance (m) x 0.0084 + 36.4.^[13]^ ECGs were recorded by a cardiologist using a Contec 1200G ECG electrocardiograph (Contec Medical Systems, China). During recording, referees were dressed in shorts, lying on their backs, relaxed, eyes closed, legs uncrossed, arms and hands flat, with electrodes placed according to standards. The skin was cleaned and dried, or waxed, to achieve optimal electrode contact. Another well-experienced cardiologist qualified in sports medicine validated and interpreted the ECGs according to the 2017 expert consensus criteria.^[8]^

### Variables studied

The independent variable studied in this study was the referee category. It was operationalised in two modalities: central referees and assistant referees.

The dependent variables studied were the quantitative and qualitative parameters of rhythm, conduction, repolarisation, and the morphology of the resting ECG associated with the athlete’s heart.^[8]^ Rhythm parameters included resting heart rate (HR), sinus bradycardia, sinus arrhythmia, and junctional escape rhythm. Conduction parameters consisted of P-wave duration (PD), PR interval (PR), incomplete right bundle branch block, first-degree atrioventricular block, and second-degree Mobitz type I atrioventricular block. Repolarisation parameters included the QT interval (QT), corrected QT interval (QTc), determined using the Bazett formula^[14]^, and early repolarisation. Morphology parameters consisted of P wave amplitude (PA), left ventricular Sokolow-Lyon index (LV-SKI), right ventricular Sokolow-Lyon index (RV-SKI), QRS axis (QRS), left ventricular electrical hypertrophy, and right ventricular electrical hypertrophy. LV-SKI was calculated from the sum of the S wave in V1 and the largest R wave in V5 or V6. RV-SKI was calculated from the sum of the R wave in V1 and the largest S wave in V5 or V6. Left ventricular electrical hypertrophy is defined as when the sum of the S wave in V1 and the largest R wave in V5 or V6 is > 3.5 mV or 35 mm. Right ventricular hypertrophy was determined when the sum of the R wave in V1 and the largest S wave in V5 or V6 is > 1.1 mV.^[8]^

### Statistical analyses

The recorded data were processed using SPSS software (IBM Statistics, Version 25). Descriptive statistics were presented as means ± standard deviations for characteristics, and as means ± standard errors of the means for quantitative ECG variables. The relative and absolute frequencies of categorical ECG variables were presented. The normal distribution of each variable was verified using the Kolmogorov-Smirnov test. The two groups of referees were compared using the Student t-test for independent samples for quantitative variables, and the chi-square (χ) test was used to compare categorical variables. The significance level for statistical tests was set at p < 0.05.

## Results

### Anthropometric and physiological characteristics of the participating referees

Except for maximal oxygen consumption, which was higher (p = 0.02) in the group of central referees, there was no difference (p > 0.05) between the groups of referees (Table 1), regardless of the characteristic considered.

### Quantitative parameter values of the referees’ resting electrocardiogram

The mean values of quantitative parameters of the resting ECG (Table 2) were higher in central referees than in assistant referees for the PR interval (p = 0.02), the QT interval (p = 0.03), and the Sokolow-Lyon index of the left ventricle (p = 0.01). They were lower in central referees than in assistant referees for resting heart rate (p = 0.04) and the corrected QT interval (p = 0.02).

### Frequency of qualitative parameters of referees’ resting electrocardiogram

Sinus bradycardia (72%), early repolarisation (44%), incomplete right bundle branch block (37%), and left ventricular electrical hypertrophy (49%) were the most common sport-related resting ECG parameters among all referees surveyed (Table 3). However, the number of referees with sinus bradycardia (23 vs. 18; p = 0.04), early repolarization (16 vs. 9; p = 0.03), incomplete right bundle branch block (10 vs. 4; p = 0.01), and left ventricular electrical hypertrophy (17 vs. 11; p = 0.02) was higher among central referees than among assistant referees (Table 3).

## Discussion

The study aimed to: 1) determine the electrical characteristics of the athlete’s heart in Burkinabe elite central and assistant football referees, and 2) compare the electrical characteristics of central referees and assistant referees. The main limitation of the study was that we were unable to include ECGs from the general population in the evaluation of ECG characteristics induced by football refereeing. The second limitation was the comparison of the results with those of athletes other than football referees. The third limitation was the small sample size, which does not allow generalisation of the results.

Sinus bradycardia, early repolarisation, incomplete right bundle branch block, and left ventricular electrical hypertrophy were the most common sport-related resting ECG changes in the referees studied. These findings support the hypothesis that the physical and physiological demands of football refereeing place a heavy burden on the cardiovascular system.^[1]^ Indeed, intense physical activity substantially increases cardiac output to meet the metabolic needs of active muscles. This causes long-term electrical manifestations on the resting ECG that reflect increased vagal tone and dilation and/or hypertrophy of the cardiac chambers.^[8]^ The surveyed referees, with an average of 6 hours of weekly training and 16 years of refereeing experience, are well beyond the minimum requirements to develop sport-related ECG changes.^[15]^ This implies that the participating referees meet the criteria for recognising electrocardiographic changes related to sports, thereby ensuring the reliability of the recorded results.

The ECG changes studied were more common among central referees than among assistant referees. The difference means that the load on the heart from refereeing is greater for central referees than for assistant referees.^[1,2]^ During a match, the average heart rate of the central referees is between 85% and 90% of the maximum heart rate (HRmax), whereas it is 76% in the assistant referees.^[2]^ An increase in heart rate increases cardiac output, better supplying the muscles with necessary nutrients, including oxygen. This is why, during a match, the average oxygen consumption increases by 70% to 80% V̇O_2_ max in the central referees compared to 65% of V̇O_2_ max in the assistant referees.^[1]^ Cardiac output can increase by a factor of 8 to reach 40 l·min^−1^, which, in the long term, represents a volume overload of the heart, resulting in electrical manifestations on the resting ECG.^[8]^

The decrease in resting heart rate and the presence of sinus bradycardia are associated with a decrease in the intrinsic beat rate of the sinus node, parasympathetic hyperactivity, and, especially, sympathetic hypoactivity.^[16]^ This reduction is not surprising given that it is widespread among athletes who train intensively.^[8]^ The frequency of sinus bradycardia observed among participating referees is similar to that reported among Burkinabe athletes at a high level of training.^[11]^ This similarity would explain the same racial origin of referees and these athletes, as ECG modifications linked to sport, such as sinus bradycardia, vary according to several factors, including racial origin, and are more common and pronounced in athletes of African ancestry than in their Caucasian counterparts.^[17]^ In athletes, sinus bradycardia is normal provided the resting heart rate is ≥30 bpm.^[15]^ It results from the normal physiological adaptation of the heart due to increased endurance training.^[15]^ It reflects the heart’s efficiency, with fewer beats needed to pump blood. However, it may indicate an underlying problem requiring medical evaluation if accompanied by intense fatigue, dizziness or fainting.^[8]^

In addition to decreased resting heart rate and sinus bradycardia, the referees presented with a prolonged PR interval and incomplete right bundle branch block. The prolongation of the PR interval and the presence of incomplete right bundle branch block could be explained by two probable factors. These are increases in vagal activity and possible morphological remodelling of the heart in response to intensive races.^[8]^ There is indeed a strong relationship between the prolongation of the PR interval, bundle branch block, and adaptations of the morphology of the myocardium.^[18]^ The frequency of incomplete right bundle branch block recorded in the referees studied is similar to that reported in high-level training athletes in Burkina Faso.^[11]^ Which means that the modifications induced by football refereeing on this parameter of conduction are similar to those caused by the practice of high-level athletics, notably endurance running. The incomplete right bundle branch block, defined as a right bundle branch block appearance with a QRS<120 ms, reflects right ventricular remodelling and does not require further examination.^[8]^

The results also showed QT interval prolongation, early repolarisation, and QTc interval shortening. The prolongation of the QT interval and the presence of early repolarisation are thought to result from prolonged cardiomyocyte depolarisation and repolarisation.^[16]^ It is noteworthy that this change is due to the autonomic nervous system’s influence on the myocardium. A decrease in resting heart rate could also explain it, as the QT interval varies inversely and proportionally with heart rate.^[16]^ The simultaneous shortening of the QTc interval is probably related to the limitations of the Bazett correction formula used. Indeed, the correction of the QT interval using this formula underestimates the QTc interval in individuals with bradycardia, specifically when the resting heart rate is less than 50 bpm.^[19]^ Electrocardiographic changes in repolarisation related to sports are mainly explained by adaptations in the density and/or activity of ion channels involved in the genesis of the action potential, as well as in the response to the autonomic balance induced by training.^[8]^ They pose the greatest difficulty in etiological diagnosis during cardiovascular assessment of athletes; therefore, their analysis must be carried out rigorously to limit errors.^[8]^ The frequency of ECG changes in repolarisation studied in referees is lower than in athletes of African origin, which could be explained by the higher level of training of these athletes.^[19]^

An increase in the Sokolow-Lyon index of the left ventricle and the presence of electrical hypertrophy of this ventricle was observed. These observations would result from remodelling of cardiac morphology in response to exercise or intensive running during matches.^[20]^ Intensive running can induce structural cardiac adaptations, characterised by left ventricular hypertrophy, which occurs in two successive phases: acute and chronic, respectively, marked by cavity dilation and ventricular changes thickening.^[20,21]^ The frequency of electrical hypertrophy of the left ventricle reported in referees was lower than that recorded in elite Ghanaian footballers, which would be at the higher training level of the latter.^[9]^ The difference could be explained by Ghanaian footballers’ higher level of training, as they are all international-level players selected for competitions organised by the International Football Association Federation. Left ventricular electrical hypertrophy is a marker of fitness, as there is a strong correlation between left ventricular hypertrophy, V̇O_2_ max, and the performance of high-level referees.^[2]^ Indeed, a high VV̇O_2_ max allows the referee to better keep pace with the game by covering more distance and repeating many sprints.^[1]^

## Conclusion

This study aimed to determine the specific electrical changes in the hearts of referees induced by football refereeing. The results showed that both central and assistant referees exhibited ECG changes in rhythm, conduction, repolarisation, and morphology associated with sport. These changes were more frequent in central referees than in assistant referees. An evaluation of the echocardiographic parameters of these two groups of referees is recommended to complement these findings.

## Figures and Tables

**Table 1:** Anthropometric and physiological characteristics of referees (n = 57)

	Total number (n = 57)	CRG (n = 28)	ARG (n = 29)	p-value

**Age (years)**	27.3 ± 2.0	26.9 ± 3.0	27.6 ± 4.0	0.74
**Height (cm)**	176.6 ± 0.1	177.1 ± 0.1	176.1 ± 0.1	0.73
**BM (kg)**	67.8 ± 4.5	68.1 ± 6.5	67.2 ± 8.7	0.58
**BMI (kg/m** **^2^** **)**	21.7 ± 1.2	21.8 ± 1.8	21.6 ± 1.9	0.69
**%BF (%)**	12.5 ± 1.4	12.5 ± 1.8	12.5 ± 1.9	0.87
**SBP (mm Hg)**	123.6 ± 4.1	125. 0 ± 5.2	122.1 ± 2.0	0.23
**DBP (mm Hg)**	78.2 ± 5.4	79.3 ± 7.3	77.1 ± 4.7	0.36
***Vdot;O*** **_2_** ** max (ml·min** **^−1^** **·kg** **^−1^** **)**	57.5 ± 1.3	58.9 ± 0.3	56.1 ± 0.4	**0.02**
**WTV (h)**	6.3 ± 1.7	6.3 ± 1.8	6.3 ± 1.6	0.99
**NYFR (years)**	17.2 ± 1.7	16.6 ± 1.9	17.9 ± 2.9	0.92

Bold indicates significance (p < 0.05). Data are displayed as means ± standard deviation. CRG, Central Referees Group; ARG, Assistant Referees Group; n, number of participants; BM, body mass; BMI, body mass index; %BF, percentage of body fat; SBP, systolic blood pressure; DBP, diastolic blood pressure; V̇O_2_ max, maximal oxygen consumption; WTV, weekly training volume; NYFR, number of years in football refereeing.

**Table 2:** Values of quantitative parameters of the referees’ resting electrocardiogram (n = 57)

	Total number (n = 57)	CRG (n = 28)	ARG (n = 29)	p-value

**HRr (bpm)**	59 ± 0.0	57 ± 0.0	59 ± 0.0	**0.04**
**PWd (ms)**	105.4 ± 1.2	105.4 ± 1.7	105.4 ± 1.5	0.83
**PWa (mV)**	0.1 ± 0.0	0.1 ± 0.0	0.1 ± 0.0	0.78
**PRi (ms)**	175.3 ± 5.4	180.7 ± 6.9	170.0 ± 8.1	**0.02**
**QRSa (°)**	59.0 ± 4.4	58.9 ± 5.3	59.1 ± 5.2	0.23
**QTi (ms)**	424.4 ± 2.0	426.8 ± 2.3	421.8 ± 2.9	**0.03**
**QTc (ms)**	412.9 ± 1.2	411.4 ± 1.8	414.3 ± 2.5	**0.02**
**ISKLlV (mm)**	28.4 ± 2.0	29.5 ± 2.2	27.3 ± 2.5	**0.01**
**ISKLRV (mm)**	8.8 ± 0.2	8.8 ± 0.6	8.8 ± 0.6	0.62

Bold indicates significance (p < 0.05). Data are displayed as means ± standard error of the mean. CRG, Central Referees Group; ARG, Assistant Referees Group; n, number of participants; HRr, resting heart rate; PWd, wave duration; PWa, wave amplitude; Pri, PR interval; QRSa, QRS axis; QTi, QT interval; QTc, corrected QT interval; ISKLLV, Sokolow-Lyon index of the left ventricle; ISKLRV, Sokolow-Lyon index of the right ventricle.

**Table 3:** Frequency of qualitative parameters of referees’ resting electrocardiograms

	Total number (n = 57)	CRG (n = 28)	ARG (n = 29)	p-value

**Sinus bradycardia**	41 (72)	23 (82)	18 (62)	**0.04**
**Sinus arrhythmia**	3 (5)	2 (7)	1 (3)	0.98
**Junctional escape rhythm**	1 (2)	1(3)	0 (0)	0.98
**Early repolarization**	25 (44)	16 (57)	9 (31)	**0.03**
**Incomplete right bundle branch block**	14 (25)	10 (36)	4 (14)	**0.02**
**1st-degree AV block**	5 (9)	2 (7)	3 (10)	0.99
**2nd-degree AV block Mobitz I**	1 (2)	0 (0.0)	1 (3)	0.95
**LV electrical hypertrophy**	28 (49)	17 (61)	11 (38)	**0.03**
**RV electrical hypertrophy**	4 (7)	2 (7)	2 (7)	0.99

Bold indicates significance (p < 0.05). Data are displayed as numbers (%). CRG, Central Referee Group; AR, Assistant Referee Group; n, number of participants; AV, atrioventricular; LV, left ventricle; RV, right ventricle.
